# Analysis of the Main Nucleosides in *Cordyceps Sinensis* by LC/ESI-MS

**DOI:** 10.3390/molecules15010305

**Published:** 2010-01-13

**Authors:** Jian-Wei Xie, Lan-Fang Huang, Wei Hu, Yun-Biao He, Kin Ping Wong

**Affiliations:** 1 Department of Chemistry and Pharmaceutical, Quzhou College, Quzhou 324000, China; 2 Changde Institute for Drug Control, Changde 415000, China; 3 Biology Department, College of Science and Mathematics, California State University-Fresno, Fresno, CA 93740, USA

**Keywords:** liquid chromatography, electrospray ionization interface, mass spectrometry, *Cordyceps sinensis*, nucleosides

## Abstract

A sensitive, selective and reliable liquid chromatography-mass spectrometry coupled with electrospray ionization interface method for simultaneous separation and determination of thymine, adenine, adenosine and cordycepin in *Cordyceps sinensis* has been established. The optimum separation for these analytes was achieved using a gradient elution system and a 2.0 × 150 mm Shimadzu VP-ODS column. 2-Chloroadenosine was used as internal standard for this assay. [M+H]^+^ions at m/z 127, 136, 268, 252 and 302 were chosen and selective ion monitoring (SIM) mode was used for quantitative analysis of the four main nucleosides. The regression equations were linear in the range of 1.0–117.5 μg·mL^-1 ^for thymine, 1.8-127.0 μg·mL^-1 ^for adenine, 0.6-114.0 μg·mL^-1 ^for adenosine and 0.5-107.5 μg·mL^-1 ^for cordycepin. The limits of quantitation (LOQ) and detection (LOD) were 1.0 and 0.2 μg·mL^-1 ^for thymine, 1.8 and 0.6 μg·mL^-1 ^for adenine, 0.6 and 0.1 μg·mL^‑1 ^for adenosine and 0.5 and 0.1 μg·mL^-1 ^for cordycepin, respectively. The recoveries of the four nucleosides ranged from 98.47 to 99.32%. The developed method was successfully used to determine nucleosides in *Cordyceps sinensis* from different sources.

## Introduction

*Cordyceps sinensis* has been used for centuries in the treatment of hyperglycemia, respiratory and liver diseases, renal dysfunction, renal failure and for its antioxidant properties [1,2]. In addition to its usage, it also has been used extensively in China as a folk tonic food or invigorant. Previous studies have found that the most important bioactive constituents in *Cordyceps sinensis* are soluble nucleosides. Because of its scarcity in Nature and high price, many fake *Cordyceps sinensis* have been found on the market, so the determination of nucleosides in *Cordyceps sinensis *is very important for its quality control [3]. Several techniques are available for the analysis of the main nucleosides in *Cordyceps sinensis*, such as thin layer chromatography (TLC) [4,5], high performance liquid chromatography (HPLC) [6,7,8,9] and capillary electrophoresis [10,11]. However, these methods can typically determine only one or two nucleosides, whose contents in *Cordyceps sinensis *are relatively high, and trace amounts of some nucleosides cannot be detected with the above methods, although these trace amount of nucleosides is also very important to the quality of *Cordyceps sinensis* from the view that its function as a traditional Chinese medicine (TCM) emphasizes its unity. In an earlier report [12], we developed a simple, fast and sensitive isocratic LC/MS method for simultaneous separation and determination of some active components in *Cordyceps sinensis*. However, thymine (a main nucleoside in *Cordyceps sinensis*) could not be determined with this method. In this paper, a simple and reliable liquid chromatography (LC)-mass spectrometric detection (MS) coupled with electrospray ionization (ESI) method for simultaneous separation and determination of the main nucleosides in *Cordyceps sinensis* is described. The developed method has successfully been used for the determination of the four main nucleosides in *Cordyceps sinensis* from various sources and can thus be used for identification, differentiation and quality evaluation of *Cordyceps sinensis*.

## Results and Discussion

### Optimization of chromatographic conditions

**Figure 1:**
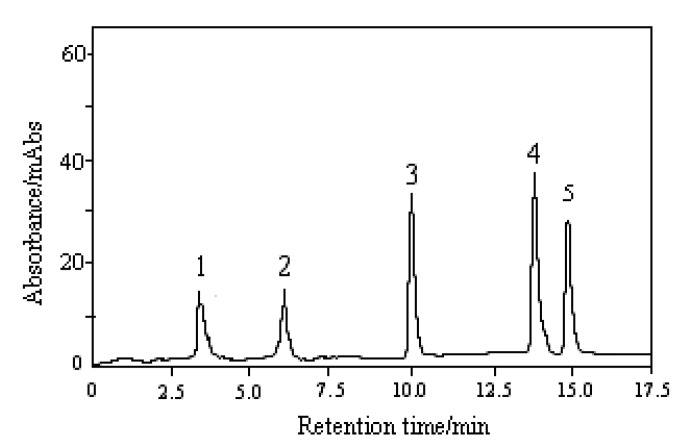
HPLC-DAD chromatogram of nucleoside standards: (1) thymine; (2) adenine; (3) adenosine; (4) cordycepin; (5) 2-chloroadenosine.

When a LC/ESI-MS method is used, the selection of mobile phase should consider both the analyte separation and the effect on ionization. Due to their similar structure the separation of nucleosides can be very difficult if an isocratic HPLC method is used. For the separation of nucleosides in *Cordyceps sinensis* and its substitutes, some reports [6] have used gradient elution with a mobile phase including (A) KH_2_PO_4_ buffer and (B) methanol, but this solvent system is not suitable for LC/ESI-MS analysis due to its low volatility and high-concentration of salts. In our study, ammonium acetate was chosen to control the acidity of the mobile phase. Using (A) ammonium acetate and (B) methanol as the mobile phase and a gradient elution program, the four main nucleosides under study were successfully separated. Chromatographic conditions, such as concentration of ammonium acetate, pH and solvent elution program, were optimized on a 2.0 × 150 mm Shimadzu VP-ODS column. The HPLC chromatogram of a mixed working standard solution and internal standard (IS) detected with a photo-diode array detector set at 260 nm is shown in Figure 1. Retention time (t_R _), UVλ_max_ values are shown in Table 1. 

**Table 1:** The values of t_R_, UVλ_max_, [M+H]^+ ^and other MS ions of nucleoside standard compounds.

Peak number	Compound	t_R_/min	UVλ_max _/nm	[M+H]^+^/m/z	[M+Na]^+^/m/z
**1**	Thymine	3.49	203, 264	127	
**2**	Adenine	5.92	207, 261	136	
**3**	Adenosine	10.12	206, 260	268	290
**4**	Cordycepin	13.75	207, 260	252	274

### Optimization of the ESI-MS conditions

**Figure 2:**
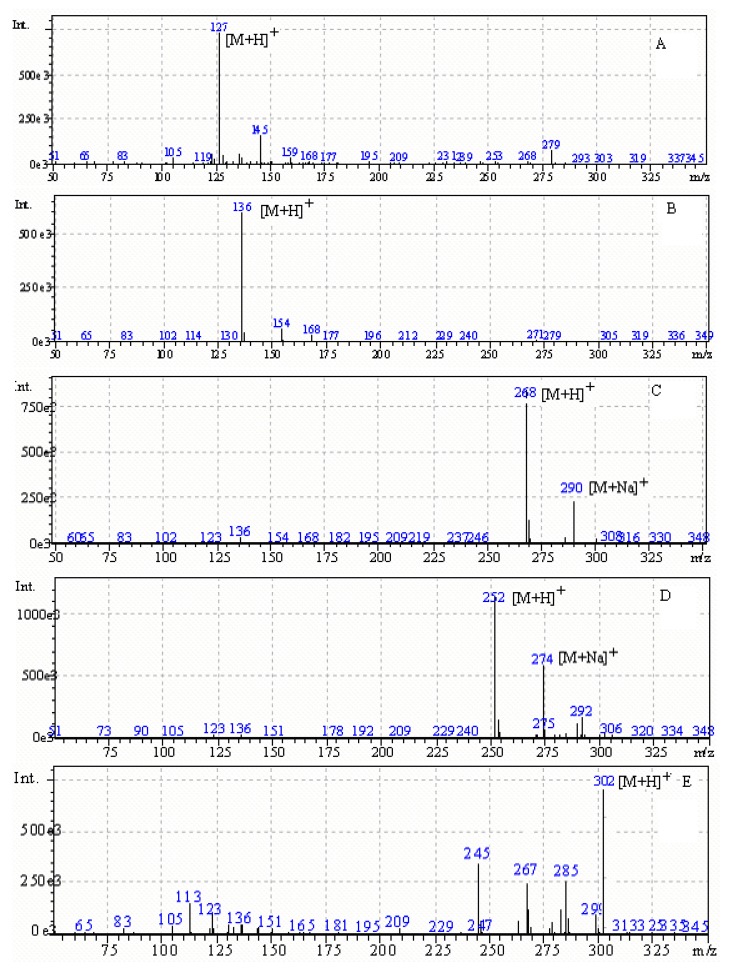
Mass spectra of nucleosides in scan mode: (A) thymine; (B) adenine; (C) adenosine; (D) cordycepin; (E) 2-chloroadenosine.

**Figure 3:**
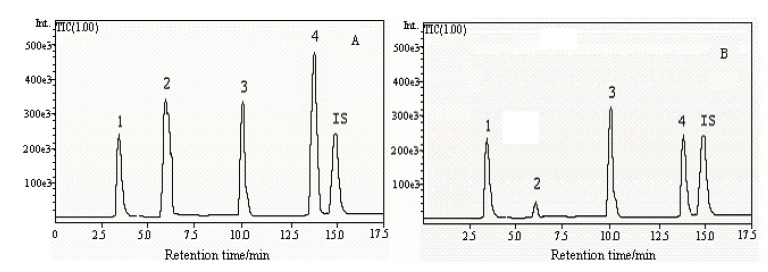
LC/ESI-MS total ion chromatograms (TIC) of nucleoside standards: (A) and water extracts of *Cordyceps sinensis *from Qinghai Province (B) (1) thymine; (2) adenine; (3) adenosine; (4) cordycepin; (5) 2-chloroadenosine.The concentration of each standard and 2-chloroadenosine was 10 µg·mL^-1^.

Both an atmospheric pressure chemical ionization (APCI) and ESI interface were tested in positive and negative ion mode by scanning between m/z 50~350 per second. The ESI interface and positive ion mode were chosen and the LC/ESI-MS mass spectra of thymine, adenine, adenosine, cordycepin and 2-chloroadenosine were obtained by scanning at the tested m/z rate of 50~350 per second. The mass spectra were shown in Figure 2. The spectra of adenine, thymine, adenosine and cordycepin were characterized by a protonated molecular ion [M+H]^+^ as base peak. In addition to the [M+H]^+^ base peak, the MS spectra of adenosine and cordycepin had a [M+Na]^+^. The results are also summarized in Table 1. SIM mode involved the use of the [M+H]^+^ions at m/z 127, 136, 268, 252 and 302 chosen for simultaneous determination of the four main active components. A mixture containing four standard compounds and 2-chloroadenosine was injected into the LC/MS system. LC/ESI-MS TIC of thymine, adenine, adenosine, cordycepin standard mixture and 2-chloroadenosine in SIM mode is shown in Figure 3A. It was observed that adenine, thymine, adenosine, cordycepin and IS were baseline resolved. Sensitivity optimization was performed by injection of an adenosine standard (20 µg·mL^-1^). 

### Method validation

A set of seven non-zero calibration standards of each component, ranging from 1.0-117.5 µg·mL^-1 ^for thymine, 1.8-127.0 µg·mL^-1^ for adenine, 0.6~114.0 µg·mL^-1 ^for adenosine and 0.5~107.5 µg·mL^-1 ^for cordycepin was prepared in order to calculate the standard curves. The calibration curves were calculated by plotting peak area ratio (Y) of each analyte and IS in LC/ESI-MS against concentrations (X, µg·mL^-1^). Each standard was analyzed in triplicate and the concentration of IS was 10 µg·mL^-1^. Results of regression equation, correlation coefficients and linear range are summarized in Table 2. 

**Table 2:** Regression equation, correlation coefficients (R), linear range and limit of detection.

Compound name	Regression equation	R	Liner range/µg·mL^-1^	Limit of detection/µg·mL^-1^
Thymine	Y = 0.06537X + 0.00614	0.9984	1.0~117.5	0.2
Adenine	Y = 0.1438X + 0.0136	0.9981	1.8~127.0	0.6
Adenosine	Y = 0.1292X + 0.0113	0.9991	0.6~114.0	0.1
Cordycepin	Y = 0.2146X + 0.0194	0.9993	0.5~107.5	0.1

The method selectivity was studied and no interference at the retention times of each analyte was observed. Because thymine, adenine, adenosine, cordycepin all exist in extracts of *Cordyceps sinensis*, no real blank was available for preparation of standards or controls. A solvent blank was analyzed and no peaks at m/z 127, 136, 268 or 252 were observed. The limits of detection of the nucleosides were in the order of 0.1–0.6 µg·mL^-1^, which were determined from signal to noise ratios of 3. Nevertheless, after applying the method to actual samples, the limits of quantification for nucleosides were considered more important. The lower limits of the range of the calibration curves can be considered as the limits of quantification of the method. The intra-assay precision and accuracy were determined by six replicate analyses of each nucleoside standards at concentrations of 2.0, 20.0 and 100.0 µg·mL^-1 ^on the same day. The intra-assay precision and accuracy results are listed in Table 3. The precisions were in the range of 1.4–6.3%. The accuracies were on the order of -3.5–4.0%. 

**Table 3:** Intra-assay precision and accuracyof thymine, adenine, adenosine and cordycepin.

Analyte	Concentration added/µg·mL^-1^	Concentration found/µg·mL^-1^	RE/%	RSD/%
Thymine	2.0	2.06	3.0	6.3
	20.0	19.7	-1.5	3.2
	100.0	101.6	1.6	1.4
	4.0^a^	3.94	-1.5	4.2
Adenine	2.0	1.93	-3.5	5.4
	20.0	20.4	2.0	3.1
	100.0	99.3	-0.7	1.6
	2.0^ a^	1.95	-2.5	2.9
Adenosine	2.0	1.96	-2.0	5.4
	20.0	20.3	1.5	3.4
	100.0	99.1	-0.9	1.7
	2.5^ a^	2.47	-1.2	3.8
Cordycepin	2.0	2.08	4.0	6.1
	20.0	20.4	2.0	2.9
	100.0	99.3	-0.7	1.9
	2.0^ a^	2.07	3.5	5.7

^a ^Aqueous extract of a *Cordyceps sinensis* sample from Sichuan spiked with thymine, adenine, adenosine and cordycepin standards.

The inter-assay precision and accuracy were determined by analyzing above samples that were tested on six consecutive days. The results of inter-assay precisions and accuracies are listed in Table 4. The inter precisions were in the range of 1.6–6.7%. The inter accuracies were in the order of -4.5–4.0%. For determination of recovery, 8.0 µg thymine, 4.0 µg adenine, 5.0 µg adenosine and 4.0 µg cordycepin were added to 100 mg *Cordyceps sinensis* from Sichuan and extracted as described under “sample preparation” in the Experimental section. This spiked sample was also used to evaluate precision and accuracy. The recoveries were 98.47%, 99.16%, 98.72% and 99.32% for thymine, adenine, adenosine and cordycepin, respectively.

**Table 4:** Inter-assay precision and accuracyof thymine, adenine, adenosine and cordycepin.

Analyte	Concentration added/µg·mL^-1^	Concentration found/µg·mL^-1^	RE/%	RSD/%
Thymine	2.0	2.08	4.0	6.7
	20.0	19.4	-3.0	4.2
	100.0	98.6	1.4	1.8
	4.0^a^	4.10	-2.5	3.8
Adenine	2.0	1.91	-4.5	6.2
	20.0	20.6	3.0	3.7
	100.0	98.7	-1.3	1.9
	2.0^ a^	2.04	2.0	5.4
Adenosine	2.0	1.94	-3.0	6.4
	20.0	20.7	3.5	3.8
	100.0	98.1	-1.9	2.1
	2.5^ a^	2.58	3.2	5.3
Cordycepin	2.0	1.92	-4.0	6.5
	20.0	20.6	3.0	3.9
	100.0	98.2	-1.8	1.6
	2.0^ a^	1.94	-3.0	6.2

^a ^Aqueous extract of a *Cordyceps sinensis* sample from Sichuan spiked with thymine, adenine, adenosine and cordycepin standards.

### Application

The developed method was used to determine nucleosides in *Cordyceps sinensis *from different sources. A representative LC/ESI-MS TIC of water extracts of *Cordyceps sinensis *from Qinghai Province in SIM mode were shown in Figure 3B. The determination results are summarized in Table 5. The results showed that there were differences between the nucleoside contents of *Cordyceps sinensis *from different sources. The contents of the four determined nucleosides obtained from different sources also varied considerably from locality to locality. Since nucleosides are important biologically active components, therefore, quality control of this commonly used drug is in great demand. The developed method could be of significant importance for the identification, differentiation and quality evaluation of *Cordyceps sinensis*.

**Table 5:** Results for the determination of main nucleosides in *Cordyceps sinensis* from different sources.

Source	Thymine/µg·g^-1^	Adenine/µg·g^-1^	Adenosine/µg·g^-1^	Cordycepin/µg·g^-1^
Qinghai	174.2	29.7	186.5	91.2
Tibet	138.5	38.1	79.6	48.7
Sichuan	142.6	20.4	126.1	31.3

## Experimental

### Apparatus

The liquid chromatography system (Shimadzu, Kyoto, Japan) consisted of an LC-10Advp solvent delivery pump, an FCV-10ALvp low pressure gradient unit, a DGU-14A degasser, a CTO-10Avp column oven, a SPD-M10Avp photodiode array detector. The mass spectrometer was a LCMS-2010 (Shimadzu, Kyoto, Japan). The column utilized for separation was a 2.0 × 150 mm Shimadzu VP-ODS column with a particle size of 5 µm. The analytical column was protected by a C_18_ Guard-pak cartridge (Waters, Milford, MA, USA).

### Materials and standards

**Figure 4:**
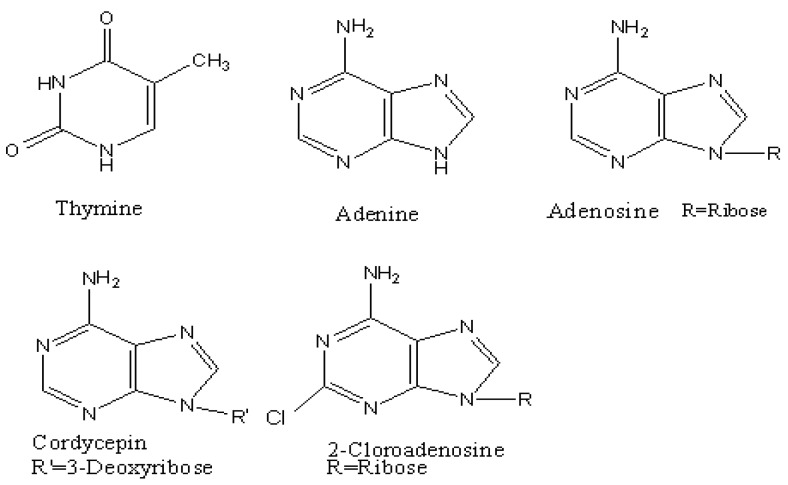
Structures of thymine, adenine, adenosine, cordycepin and 2-chloroadenosine.

Standards of thymine, adenine, adenosine, cordycepin and 2-chloroadenosine (their structures are shown in Figure 4) were purchased from Sigma (St. Louis, MO, USA), A stock solution of each standard (0.2 mg·mL^-1^) was prepared in methanol and stored in a refrigerator. Dilutions of 2.0 and 20.0 µg·mL^-1 ^of each compound were prepared by diluting the stock solution with methanol. Acetic acid and ammonium acetate used were analytical grade. HPLC-grade methanol was obtained from Hanbang Science and Technology CO. (Jiangsu, China) and Milli-Q quality water was used in the preparation of the mobile phase. Solvents were filtered through a 0.45 µm membrane and degassed. *Cordyceps sinensis* from Qinghai, Sichuan and Tibet Autonomous Regin of the P.R. China was purchased from local herb stores in Changsha.

### Sample preparation

*Cordyceps sinensis* was dried at 35 °C for two hours and first ground into powder (approximately 20 mesh). Subsequently, it was weighed (about 100 mg) and added to distilled water (20 mL) for extraction of nucleosides. The sample-water mixture was placed into an ultrasonic bath for two hours. Then the sample-mixture was filtered and the filtrate was vacuum-dried. The residue was dissolved in methanol (2 mL).

### Chromatographic conditions

The mobile phase including (A) ammonium acetate (40 mM, pH = 5.2) and (B) methanol was degassed ultrasonically before use. Each component of the mobile phase was filtered through a 0.22 µm membrane. Chromatographic separation was performed at room temperature and a flow-rate of 0.2 mL/min. The solvent elution program was shown in Table 6. The wavelength of the photo-diode array detector was 190~300 nm. The injection amount was 5 µL.

**Table 6:** Solvent elution program for HPLC.

Step	Time/min	Ammonium acetate (40 mM, pH=5.2)/%	Methanol/%
1	0	85	15
2	6	85	15
3	12	80	20
4	17	80	20
5	20	95	5

### Mass spectrometric detection conditions

MS coupled with an electrospray ionization (ESI) interface in both scan and SIM mode was used. [M+H]^+^ at m/z 127, 136, 268, 252 and 302 (IS ion) was selected as the SIM ions for qualitative and quantitative analysis. The ESI temperature was 400 °C. The curved desolvation line (CDL) and block temperature were 250 °C and 200 °C, respectively. Probe voltage was +4.5 KV. Detector voltage was 1.5 KV. CDL Voltage was –20 V. Q-array Bios was 50 V. Nebulizing gas flow was 4.5 L/min.

## Conclusions

By use of a gradient elution system and 2-chloroadenosine as internal standard, a suitable LC/ESI-MS method for simultaneous separation and determination of the four main nucleosides in *Cordyceps sinensis* has been established and validated. The developed method can be used for the identification, differentiation and quality evaluation of this material. The obtained results show that the LC/ESI-MS method is a powerful technique for the analysis of bioactive components in TCMs.
